# The Stroop Task Sex Difference: Evolved Inhibition or Color Naming?

**DOI:** 10.1007/s10508-022-02439-9

**Published:** 2022-10-19

**Authors:** Espen A. Sjoberg, Raquel G. Wilner, Antonia D’Souza, Geoff G. Cole

**Affiliations:** 1 School of Health Sciences, Kristiania University College, Prinsens gate 7-9, 0152 Oslo, Norway; 2 Department of Linguistic, Literary and Aesthetic Studies, University of Bergen, Bergen, Norway; 3 Centre for Brain Science, University of Essex, Colchester, UK

**Keywords:** Inhibition, Stroop task, Negative priming, Evolved inhibition hypothesis, Verbal abilities, Sex/gender differences

## Abstract

Previous research shows that women outperform men in the classic Stroop task, but it is not known why this difference occurs. There are currently two main hypotheses: (1) women have enhanced verbal abilities, and (2) women show greater inhibition. In two Stroop experiments, we examined the Inhibition hypothesis by adopting a procedure, often used in visual cognition paradigms, that induces a particular inhibitory component. So-called Negative Priming occurs when a distracting non-target stimulus on one trial becomes the target on the following trial. Results from our experiments showed that the degree to which this type of inhibition occurs within the Stroop effect is no different for men and women. This was the case irrespective of whether participants made a vocal response (Experiment 1; *n* = 64, 32 men and 32 women) or a manual response (Experiment 2; *n* = 64, 32 men and 32 women). These results do not therefore support the Inhibition hypothesis. We additionally review findings from a range of paradigms that can be seen as indexing the different components required for the Stroop task (e.g., distractor suppression). This review suggests that the sex effect is due to superior color naming ability in women.

## Introduction

The Stroop (1935) task is arguably the most well-known procedure in cognitive psychology. In the standard experiment, participants are asked to verbalize the ink color of a word as quickly as they can. Even though the procedure has multiple features, the term “Stroop” typically refers to the Color-Word component of the task, or what is most often referred to as the “incongruous” condition. This involves the presentation of color words in conflicting ink colors (e.g., the word “red” displayed in blue ink), and the participant is asked to ignore the written word and name the ink color. Time to determine ink color is typically longer in this situation compared to when there is no conflict (e.g., the word “red” written in red ink). In the 85 or so years since the original Stroop paper, the procedure has been employed in a variety of applications throughout several fields. For instance, it has been used to assess cognitive functioning in participants with autism (Adams & Jarrold, 2009), schizophrenia (Aksoy-Poyraz et al., 2011), and multiple sclerosis (Scarrabelotti & Carroll, 1999).

### Sex Differences in the Stroop Task: Verbal Abilities or Inhibition?

In his extensive review of the Stroop phenomenon, MacLeod (1991) wrote: “Here is a summary statement: There are no sex differences in Stroop interference at any age. Perhaps this is too strong, but I remain to be convinced” (italics removed).^1^ This conclusion was, however, only based on a dozen reports. Furthermore, several studies published since the MacLeod review have now reported a significant reaction time advantage for women across a variety of cultures and age groups, (e.g., Buck, Hillman, & Casteli, 2008; de Grip et al., 2008; Laeng et al., 2005; Pati & Dash, 1990; Seo et al., 2008). Because of the large sample size, the van der Elst et al. (2006) study is particularly worthy of note. Here, van der Elst et al. observed “clear sex differences” with an advantage in women amongst 1856 Dutch adults. Sjoberg and Cole (2022) have also recently completed a meta-analysis on every Stroop experiment published (and some unpublished) from 1935 to 2013 in which sex differences can be determined on the Color-Word (i.e., incongruous) task. Across 126 experiments, Sjoberg and Cole found a small but highly significant advantage in women (*d* = 0.12, *p* < .0001). It is also worth noting that not only did Stroop (1935) find an advantage in women, a superiority effect in men is almost never observed (Asha, 1989; von Kluge, 1992).

When a sex effect is found, the most commonly proposed explanation is that women have superior verbal abilities compared to men (e.g., Lee et al., 2004). As outlined by von Stumm et al. (2020), such abilities include knowledge, proficiency, and fluency of grammar, vocabulary, words, opposite analogies, phonological awareness, and articulation (see Roivainen, 2011, for a review). These can be contrasted with non-verbal abilities which include puzzle solving, numeracy, spatial processing, and drawing. With respect to the Stroop effect, the specific argument is that women are able to access color labels more rapidly than men (Golden, 1974; Lee et al., 2004; MacLeod, 1991; Seo et al., 2008). Indeed, this explanation was proposed by Stroop (1935) who cited Brown (1915) and Ligon (1932) as evidence. The color naming hypothesis is also supported by the observation that women often show a superiority effect on the color naming control task that is sometimes included in the Stroop paradigm (e.g., Jensen & Rohwer, 1966; Strickland et al., 1997). Here, participants are typically asked to name the color of a square patch as fast as they can.

An alternative to the verbal abilities hypothesis was proposed by Bjorklund and Kipp (1996). In a broad and extensive review of sex differences in inhibition, the authors argued that women could have evolved superior inhibition mechanisms as a result of differential mating strategies. In short, females in most species invest greater resources in their offspring (e.g., gestation) and can therefore be more selective when it comes to choosing a mate (Trivers, 1972). This could manifest as greater inhibition of behavior in contexts related to reproduction in order to avoid choosing or sending signals to a potentially unsuitable mate. By contrast, males need no such inhibition mechanism because their best strategy is to be indiscriminative when choosing a female (Janetos, 1980). This hypothesis was subsequently named the Female Evolved Inhibition hypothesis (Sjoberg & Cole, 2018).

As reviewed by Bjorklund and Kipp (1996), inhibition in women is particularly strong in social contexts related to sexual behavior. For instance, women are better at inhibiting their sexual arousal (Chivers et al., 2010; Milhausen et al., 2010). Even if women are as implicitly aroused as men they express less explicit arousal (Suschinsky et al., 2009). Whatever the reason for the greater propensity for inhibition behavior, the Bjorklund and Kipp review showed that the inhibition effect occurs on a large range of tasks measuring, what they called, social inhibition (e.g., emotion and arousal oriented behaviors) and behavioral inhibition (i.e., temptation, delayed gratification, impulse control). Bjorkland and Kipp put forward a “generalizability” hypothesis in which they suggested that the neural circuits that generate these inhibitory behaviors could be used in other (“cognitive”) tasks that do not possess any social or behavioral component. One example would be the visuo-motor phenomenon known as “inhibition of return” in which observers are relatively slow to process stimuli located at a position recently attended (Posner & Cohen, 1984). Although the task only requires the pressing of a button in response to a simple target, Brown (2013) showed that this particular inhibition effect is larger in women.

### The Negative Priming Stroop Task

Another non-social visual cognition inhibition phenomenon is the Negative Priming effect (Tipper, 1985) or Distractor Suppression effect (Neill, 1977). Here, the design of the paradigm includes a trial sequence in which a non-target distracting stimulus on trial *N*-1 is the target on trial *N*. For example, a participant may be required to identify words that are placed within (to-be-ignored) pictures. On Trial *N*-1, the (irrelevant) image could be a dog and the target word is “Dog” on the following trial. In this scenario, reaction times are typically longer compared to when the image on trial *N*-1 was, for instance, a horse. The argument is that the suppression of a distractor that is induced on one trial carries over to the following trial. This procedure is thus believed to isolate one particular inhibitory component (Neill & Westberry, 1987; Tipper et al., 1989).

In the Stroop variant of this paradigm, the to-be-ignored color on one trial is the to-be-named color on the following trial. For example, trial *N*-1 might present the word “Red” in blue ink (i.e., correct response is “Blue,” distractor is “Red”) and trial N presents the word “Green” in red ink (i.e., correct response is “Red,” distractor is “Green”). There currently exist only four Negative Priming Stroop studies that have included sex as a variable of interest. In three of these, no significant differences were found in Negative Priming (Christiansen & Oades, 2010; Claridge et al., 1992; Steel et al., 1996). The fourth study found a significant advantage in women (Visser et al., 1996). This, however, was based on overall performance of children with varying levels of cognitive and social impulsivity.

### The Present Experiments

The central aim of the present study was to examine the inhibition hypothesis with respect to the Stroop advantage observed in women. In both our experiments, participants performed a Stroop procedure that included Color-Word (“incongruous”) and Negative Priming conditions. Any overall difference (i.e., irrespective of sex) in performance between these two can be taken to reflect the additional inhibition component induced by the Negative Priming procedure. If the advantage in women on the Stroop task is due to increased inhibition, one might expect a sex by trial type interaction. Specifically, women should exhibit greater Negative Priming relative to men.

## Experiment 1

Participants were presented with color words, rendered in one of five colors, and were asked to state the ink color aloud. The words were presented as a string of text within a number of sequential displays.

### Method

#### Participants

Participants were 64 adults of varying nationality and ranging in age from 18 to 73. There were 32 men (mean age 33.2) and 32 women (mean age 30.8). They did not significantly differ in age, *t*(62) = 0.64, *p* = .51.

#### Stimuli

A total of 900 colored words were presented in total by showing 30 displays each containing 30 words. Given how robust the basic Stroop phenomenon is (when participants vocally respond to a variety of colors), we did not include the congruent condition. Thus, all words were printed in an incongruent color. These (and their respective Red, Green, and Blue, “RGB color space” values) were RED (255, 0, 0), BLUE (0, 93, 162), GREEN (7, 169, 11), WHITE (255, 255, 255), and BROWN (102, 51, 0). The words were in the font Times New Roman, size 46. Each appeared five times on every display, and each ink color occurred six times. The order of the words was randomized, as was the ink color. However, after this randomization, displays were manually corrected to ensure that there were no: (1) congruent words, (2) color-word combinations or ink colors repeated consecutively, and (3) negative priming items in the standard Stroop Color-Word task. All stimuli were presented on a 17-inch computer monitor and presented via PowerPoint.

Fifteen of the (30-word displays) were comprised of Color-Word trials, and the other 15 Negative Priming trials. The 15 trials showed high reliability with both Color-Word, *α* = 0.98, and Negative Priming, *α* = 0.98.

#### Design and Procedure

A 2 × 2 mixed design was employed with sex as the between-participants factor and trial type (Incongruous, Negative Priming) as the within-participants factor. The dependent variables were the time taken to name all the colors in each 30-word display (measured in seconds and centiseconds), the number of errors made but corrected by the participant (corrections), and the number of errors made that were not corrected (uncorrected errors). Time taken was defined as the time from stating the color of the first word to the final word in each display. This was done in real time and logged by the experimenter. The experimenter had a copy of the correct answers for each display and noted whenever a correct or uncorrected error was made. Reaction times and errors were decoded from these sheets at a later time. Inter-rater reliability was not measured, since this would mostly affect the error rates, which was a secondary measure.

Prior to the experiment, participants indicated by self-report that they had no color vision deficits. They were instructed to name the ink color of the printed words as quickly and as accurately as possible. Before the first display was presented, they were given 10 Color-Word practice trials. Participants were told to correct their response if they were aware that they had made an error. Reaction time was measured by the experimenter using a digital stopwatch. Participants were offered a break every 10 displays. Display order was randomized.

### Results

**Fig. 1:**
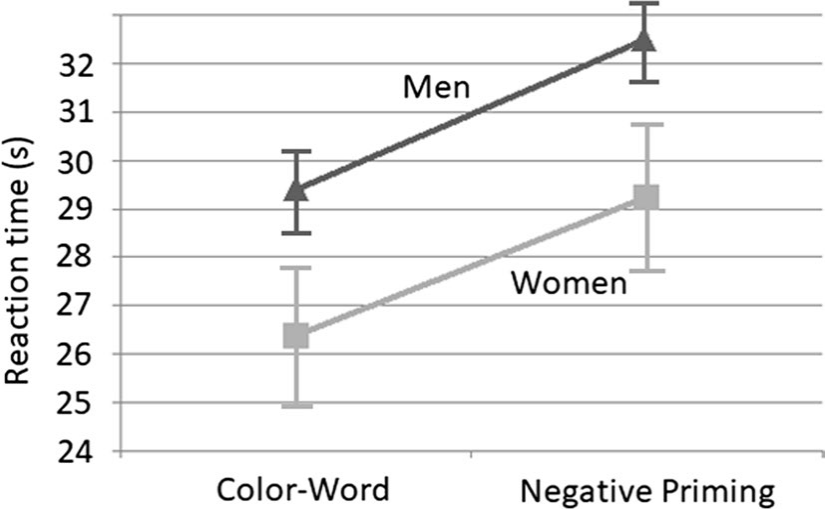
Mean reaction times and standard errors in Experiment 1. Note that *n* = 32 women and 32 men

One participant was removed from further analysis because he was unable to distinguish between the colors green and brown, despite reporting no color deficits (he made uncorrected errors more than five standard deviations above the mean). For the remaining 63 participants, a mean reaction time was calculated for the Color-Word and Negative Priming trials. These are shown in Fig. 1.

With respect to reaction time, a 2 × 2 mixed ANOVA with sex as the between-participants variable and trial type as the within-participants variable found a significant main effect of trial type, *F*(1, 61) = 154.13, *p* < .001, *η*_p_^2^ = 0.716. A main effect of sex was also found, *F*(1, 61) = 4.16, *p* < .05, *η*_p_^2^ = 0.064. There was no significant interaction, *F*(1, 60) = 0.33, *p* > .56, *η*_p_^2^ = 0.005. In terms of errors, there was no sex difference for either corrected means (Women = 10.2, *SD* = 6.8, Men = 10.2, *SD* = 8.4; *t*(62) = 0.02, *p* > .98) or uncorrected (Women = 4.1, *SD* = 8.4, Men = 3.3, *SD* = 3.0; *t*(62) = 0.5, *p* > .62). Because of the large age range, we were also able to assess performance as a function of age. We found that age correlated significantly with performance in both the Incongruent, *r* = .39, *p* < .002, and the Negative Priming conditions, *r* = .30, *p* < .02, suggesting that performance decreased (higher reaction time) as age increased. Age did not correlate with number of corrections, *r* = .14, *p* = .286, or uncorrected errors, *r* = − .04, *p* = .759.

Overall, these results show a large Negative Priming effect. Participants were slower to respond when the target had been the distractor on the proceeding trial. Importantly, however, is the observation that this was no different for men and women. These data do not therefore support the inhibition account.

## Experiment 2

As a further assessment of the inhibition hypothesis, we developed a two-alternative, manual response, forced-choice, variant of the Stroop paradigm. On each trial, participants were presented with a single word (“RED” or “GREEN”) located in the center of the display. The word would be written in either red or green ink, and participants were required to indicate the ink color with a button press. A single block of trials included congruent and incongruent conditions with the trial order also generating a Negative Priming condition within the latter. For instance, trial *N*-1 could present the word RED written in green ink and trial *N* would sometimes be the word GREEN written in red ink.

### Method

#### Participants

A total of 65 (32 men, 33 women) adults took part. All were aged either 19 or 20 years and were enrolled onto a Psychology undergraduate degree at Essex University. None had taken part in Experiment 1.

#### Stimuli

Two possible words could appear, RED or GREEN. These were either written in red ink (Red, Green, Blue color space values being 237, 28, and 36, respectively) or green ink (Red, Green, Blue being 34, 177, and 76). This generated four possible trial types. On a 32 × 20 cm monitor, the letters comprising each word were 15 mm in height and 10 mm in width. The font was Ariel Bold.

#### Design and Procedure

We employed a 2 × 2 mixed design with sex (men, women) as a between-participants factor, and trial type (Incongruous, Negative Priming) as a within-participants factor. The dependent variable was time to indicate color ink via button press for each word presentation. A total of 192 trials were presented in a single block. The four trial types were presented in a pseudo-random, rather than random, order to ensure Negative Priming trials were generated. The experiment was run using PsychToolKit (Stoet, 2010), a well-established experimental platform that supports millisecond timing precision. The task was undertaken “remotely.” Thus participants accessed the experiment via a web-link on their own device. The link directed them to a general introduction display that provided general background about the experiment (e.g., ethics information and approval number) and stated that the experiment required a keyboard. Instructions asked participants to indicate the ink color of each word as quickly as they could by pressing the Z button with their left index finger for “red” and M button with their right index finger for “green.” They were asked to rest these two fingers on the two keys. The design of the experiment, including participant number was preregistered at https://aspredicted.org/my5fe.pdf. This preregistration also presents details of the analysis including the criteria for reaction time outliers.

#### Data Preparation and Initial Analysis

Around 40 participants indicated that they agreed to take part but then did not do so. This may be because initially they didn’t realize that a keyboard was necessary. One (woman) participant who did complete the experiment did not achieve the required 80% color discrimination accuracy and was replaced. Data from 32 men and 32 women were thus analyzed. We are not aware of any previous Stroop experiment that only employed two colors and two manual responses. Our preregistration therefore stated that we would initially examine whether a basic Stroop effect occurred before we conducted the central analysis regarding any sex and inhibition effect. Reaction times shorter than 200 ms and longer than 5 s were omitted. This resulted in 67 trials being excluded across all participants. A significant Stroop effect was found (Congruent mean = 506, SD = 90, Incongruent mean = 548, SD = 119), *t*(63) = 7.1, *p* < .00001.

### Results

**Fig. 2:**
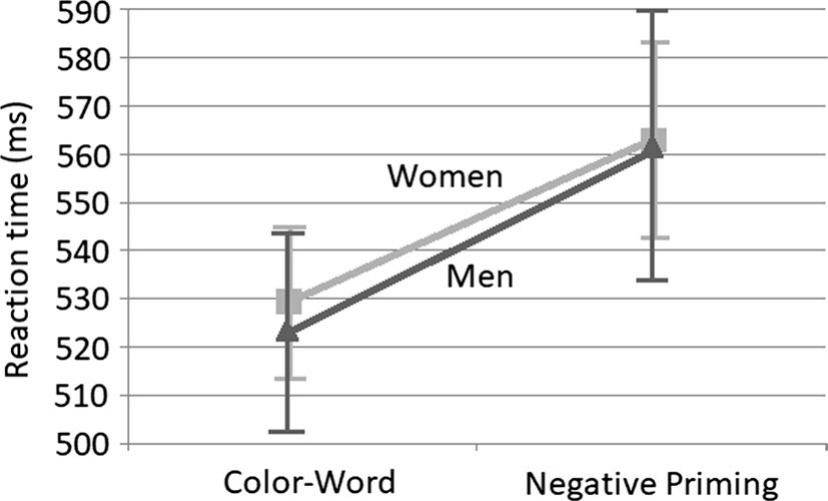
Mean reaction times and standard errors in Experiment 2. Note that *n* = 32 women and 32 men

Figure 2 shows mean reaction times and error rates together with standard errors for all four conditions. A 2 × 2 mixed ANOVA revealed a significant main effect of trial type, *F*(1, 62) = 23.6, *p* < .001, *η*_p_^2^ = 0.28, but no such effect of sex, *F*(1, 62) = 0.01, *p* > .91, *η*_p_^2^ = 0. The interaction was not significant, *F*(1, 62) = 0.1, *p* > .75, *η*_p_^2^ = 0.002. In terms of percentage error rates, a significant main effect of trial type was observed, *F*(1, 62) = 73.00, *p* < .001, *η*_p_^2^ = 0.54, (Incongruous = 11.0%, *SD* = 6.6, Negative Priming = 5.0%, *SD* = 6.0) but no effect of sex, *F*(1, 62) = 0.30, *p* > .59, *η*_p_^2^ = 0.005, (Women = 8%, *SD* = 7.4, Men = 7.5, *SD* = 5). There was a non-significant interaction, *F*(1, 62) = 3.42, *p* > .07, *η*_p_^2^ = 0.051.

Subsequent to our pre-registration, we became aware of Mills et al. (2019). These authors suggested that reports of the Negative Priming effect may actually be due to an experimental confound. Negative Priming trials always present a target that was a non-target on the previous trial. In other words, Negative Priming trials never repeat a target from trial-to-trial. The Negative Priming condition is then typically compared with a condition that does include repeated trials. Since reaction time for targets that happen to repeat are particularly short (see Hillstrom, 2000), data can therefore look like a Negative Priming effect when the real effect is no more than an intertrial repetition effect. We therefore undertook an additional analysis in which the (non-Negative Priming) incongruent condition only included trials that did not involve a repeating target. Results again revealed a significant main effect of trial type, *F*(1, 62) = 10.11, *p* < .002, *η*_p_^2^ = 0.14, and no effect of sex, *F*(1, 62) = 0.08, *p* > .78, *η*_p_^2^ = 0.001. The interaction was not significant, *F*(1, 62) = 0.25, *p* > .61, *η*_p_^2^ = 0.004. Thus, our Negative Priming effect is not a consequence of repeated targets. As with our primary analysis for both experiments, these data, including trial order and the trials included in this analysis, can be seen at https://osf.io/qwpaf/.

In sum, Experiment 2 again failed to support the inhibition hypothesis. Women did not show greater Negative Inhibition on the Stroop task.

## General Discussion

In two experiments, we have examined the inhibition account of the reaction time advantage for women in the Stroop effect. Our two experiments were designed to induce a particular type of inhibition within the basic paradigm, i.e., Negative Priming. Although we observed this phenomenon in both experiments, the effect was no different for either sex. These results do not therefore support the inhibition account. We contend that the Stroop advantage in women is instead due to some aspect of superior verbal ability, an effect often reported. The question is which of the various verbal abilities is responsible?

In the present Introduction, we noted that “verbal abilities,” although often employed in the literature, is a broad term that includes a range of processes concerned with some aspect of language (e.g., grammar, vocabulary, naming). Indeed, Roivainen (2011) reflected how broad the notion is when he referred to the advantage in women as being concerned with “digits and alphabets.” If one considers the more specific aspects of this umbrella term, the consensus has consistently stated that women outperform men on a range of tasks. These have included object naming (Camarata & Woodcock, 2006), phonological coding (Majeres, 1983, 1988), reading speed (Halpern, 2000; Lynn & Mikk, 2009), and handwriting speed (Graham et al., 1998; Tseng & Hsueh, 1997). Some of these abilities are not of course involved in successful Stroop performance. This is clear when we consider the specific kind of task used to measure each. For instance, the “reading fluency” component of the commonly used Woodcock-Johnson-III test of general intellectual ability is defined as “reading printed statements rapidly and responding true or false (Yes or No)”. This clearly involves comprehension and some aspect of logical reasoning both of which are not needed for the Stroop task. In a review article, Wallentin (2009) noted that the verbal fluency task is perhaps the most cited verbal ability procedure that has yielded sex differences (e.g., Sommer et al., 2004). In the standard version of the task, participants are required to generate as many words as they can from a particular category (e.g., animals), or beginning with a certain letter, within a given time (e.g., five minutes). Again, this type of verbal ability is irrelevant to the Stroop procedure.

Rather than simply attribute the advantage in women to the vague notion of “superior verbal abilities,” a better strategy for identifying the cause of the sex effect is to consider the abilities that are required for Stroop performance and assess whether any sex differences occur within these. A reasonable assumption is that participants need to recognize and discriminate the stimulus color, “find” the correct label, and then execute the response. Other mechanisms are of course involved that include, for instance, “early” visual processes that generate the experience of color. However, it is unlikely that much variance in Stroop responses can be attributed to processes other than discrimination, naming, and response. There is also the important consideration of needing to ignore the irrelevant stimulus.

Below, we outline the four specific processes involved in Stroop performance (color discrimination, color naming, distractor suppression, response execution) and assess which shows an advantage in women.

### Color Discrimination

In terms of the first process, many authors argue that the evidence for an advantage in women on color recognition/discrimination tasks is at best mixed. Over fifty years ago, Reynolds (1966) attempted to trace the history of what he concluded was a “scientific fiction”. Despite it being mentioned in at least one influential text book (Klineberg, 1949), Reynolds could find no studies supporting the assertion. For instance, during the development of a color discrimination test, Pickford (1951) tested 1100 participants and found no sex effect. Similarly, Verriest et al. (1962) found no overall difference in almost 500 men and women of different ages using the Farnsworth-Munsell 100-hue test. Based on discussions with colleagues, Reynolds concluded that the basis for the “usubstantiated claim” was in the then common notion that there is “greater feminine concern with matters of dress and décor” (p. 85). More recently, Hood et al. (2006) also found no sex differences (in a control group for an X-linked color deficiency group) and referred to the “entrenched belief that women enjoy better color discrimination than do men” (p. 2898). It is possible that the notion has arisen because color deficiency is well-known to affect men more than women.

One also has to note that studies of color discrimination use extremely sensitive procedures (e.g., the Farnsworth-Munsell 100 Hue Test) that are designed to detect differences between groups and conditions for colors that are very close together in any given color space. No such level of color discrimination ability is typically required for the Stroop task. Participants are usually required to discriminate between five or six colors only; colors that are relatively far apart in color space. Responses are effectively at ceiling in terms of stimulus recognition.

We posit that processes involved in color recognition and discrimination are not responsible for the Stroop advantage in women.

### Color Naming/Labeling

In contrast to color discrimination, color labeling studies have consistently found a sex difference using a range of paradigms. For instance, women tend to use a wider range of color terms (Lin, Luo, MacDonald & Tarrant 2001; Nowaczyk, 1982; Rich, 1977; Swaringen et al., 1978; Thomas et al., 1978) and are more accurate in ascribing color names to colored stimuli (Green & Gynther, 1995). Most closely related to the Stroop procedure, however, are tasks in which participants make a speeded response. Here, women have unequivocally been shown to outperform men in terms of how quickly they can access a color label when presented with a colored stimulus (DuBois, 1939; Golden, 1974; Jensen & Rohwer, 1966; Ligon, 1932; Saucier et al., 2002; Shen, 2005; Strickland, et al., 1997). Interestingly, although there are reports of an absence of a color naming advantage in women (e.g., Rognoni et al., 2013), there are no reports of men outperforming women.

It is also illustrative to compare this effect with that of sex differences in reading speed. Many Stroop procedures, particularly those published within the first decades of Stroop’s original paper, presented the words as strings of written text, as in the present Experiment 1. This inevitably involves processes associated with reading, another verbal-type skill often reported as being superior in women (e.g., Camarata & Woodcock, 2006). A number of studies have examined sex differences in color labeling speed and reading speed within the same study and often the same participants. In one of the first (non-Stroop) studies, Ligon (1932) asked 638 school children from 9 different grades to read the names of 100 color words (all presented in the same ink color) and in a second task state the color of 100 square patches (with presentation order of the two tasks being counterbalanced). While an advantage in females was observed for color naming, no such effect occurred for word reading. The van der Elst et al. (2006) study of 1856 Dutch adults also found no sex effect in reading color words but did so for naming colors. The differential effect of naming and reading has been reported on a number of occasions (e.g., Baroun & Alansari, 2006; Oosthuizen & Phipps, 2012; Sladekova & Daniel, 1981).

In sum, women have consistently been found to outperform men in terms of how fast they can access color labels.

### Distractor Suppression

The third component of Stroop performance is the ability to ignore the irrelevant stimulus during incongruent trials. As Coleman et al. (2018) stated, “multiple studies” have reported longer reaction times and/or reduced accuracy in women on paradigms that include irrelevant conflicting stimuli. For example, Stoet (2010) used a variant of the Flanker procedure (Eriksen & Eriksen, 1974) in which a centrally located target is flanked by irrelevant distracting stimuli. In the Stoet variant, the target was a green disk that acted as the “GO” stimulus and a red disk indicated that a response should be withheld. The irrelevant flankers were either green, red, or blue discs. Stoet found that women were significantly slower and made more errors (i.e., false starts) on incongruent trials (i.e., red flankers). Importantly, there was no difference on congruent trials (i.e., green flankers) and neutral trials (i.e., blue flankers). This shows that the advantage in men was not because they were faster overall; men were less distracted by conflicting information. The same pattern of data was observed in a similar experiment using targets and distractors that were semantically related (Judge & Taylor, 2012). In sum, men are better than women at ignoring distracting stimuli.

### Response Execution

This process occurs when all the appropriate decisions have been made and the participant has to execute a response. Paradigms most pertinent to this issue are ones in which participants are asked to make a single speeded response to a stimulus that requires (relatively) minimal cognitive processing, which is sometimes called simple reaction time. For instance, a participant may be asked to respond whenever they see the onset of the digit “0”; no other stimulus appears and they are required to press one button with one finger. Using this paradigm, and re-analyzing a data set from over 7000 adults, Der and Deary (2006) found a significant advantage in men (eta squared = 0.12). This concurs with the meta-analysis of Silverman (2006) who found a Pearson’s *r* of 0.17 derived from 21 studies (*n* = 15,003) published over a 73-year period. These large data sets clearly suggest that women do not show a simple reaction time advantage. Thus, the Stroop sex effect is unlikely to be due to this late response process.

### Color Naming as the Locus of the Stroop Sex Effect

The above brief review shows that on three of the four processes directly relevant to the Stroop task (i.e., discriminating color, ignoring irrelevant information, and response execution) women do not outperform men. Indeed, on two of these (response execution and ignoring irrelevant information) men outperform women. It follows therefore that the reason for the sex effect is because of the one process in which women are superior, that is, color naming. This hypothesis is also supported by the flanker experiments described above. These procedures are conceptually similar to the Stroop paradigm in the sense that a target is accompanied by distracting information. However, color naming was not part of those flanking procedures. Consequently, no advantage in women was observed.

Although the present experiments were specifically designed to assess the inhibition hypothesis, our data may also provide support for the color naming hypothesis. It is likely that color naming processes were involved to a greater degree in Experiment 1 compared with Experiment 2, whereas the latter only employed two colors the former employed five. Color labeling would therefore have been more important to successfully perform the task in Experiment 1. Indeed, it would be difficult to perform without access to labels, which of course helps a participant to remember the mapping between the stimulus and response required. This was not the case for Experiment 2 where only two colors were employed. In fact, a sample of participants who, for some reason, never learned to label colors would still be able to perform our second study. In this thought-experiment, these participants would not need language to discriminate between the two stimuli. They would just need to know that one stimulus (what everyone else labels as “red”) requires a left button response and the other stimulus (what everyone else labels as “green”) requires a right response. This could be taught to such participants within moments.

Finally, it is interesting to consider why women have superior color naming ability. One explanation is that it is a consequence of superiority in general color fluency and use. For example, women are known have a larger color vocabulary (Nowaczyk, 1982; Simpson & Tarrant, 1991), and when asked to create a drawing, women use a larger range of colors (Wright & Black, 2013). Effects such as these could in turn reflect the fact that women are more familiar with a wider range of colors. This could occur via advertising. As Koller (2008) found, there are color differences in products marketed at men and women. It is also possible that the color naming advantage in women is due to superiority in being able to access any label from memory, irrespective of what category the label comes from (e.g., colors, animals, faces). Indeed, in a sample of over 2000 individuals, Camarata and Woodcock (2006) found that women produced shorter reactions on the Rapid Picture Naming component of the Woodcock-Johnson-III test.

In sum, the present experiments have found no evidence for the evolved inhibition hypothesis of sex differences in the Stroop task. This together with previous work suggests that the sex effect is due to the fact that women exhibit superior color naming ability.

## Data Availability

Available at https://osf.io/qwpaf/.

## Footnotes

1 We adopt the American Psychological Association’s advice on the use of gendered/sex terms (see https://apastyle.apa.org/style-grammar-guidelines/bias-free-language/gender). For instance, “men” and “women” are used rather than “male” and “female.”
